# A blended face-to-face and smartphone intervention to improve suicide prevention literacy and help-seeking intentions among construction workers: a randomised controlled trial

**DOI:** 10.1007/s00127-023-02429-9

**Published:** 2023-02-09

**Authors:** Tania L. King, Ludmila Fleitas Alfonzo, Philip Batterham, Andrew Mackinnon, Chris Lockwood, Samuel Harvey, Brian Kelly, Helen Lingard, Laura Cox, Tony D. LaMontagne

**Affiliations:** 1 Centre for Health Equity, Melbourne School of Population and Global Health, The University of Melbourne, Melbourne, 3010 Australia; 2 Australian National University, Canberra, Australia; 3 University of New South Wales, Sydney, Australia; 4 MATES in Construction, Spring Hill, Australia; 5 University of Newcastle, Newcastle, Australia; 6 RMIT University, Melbourne, Australia; 7 Deakin University, Geelong, Australia

**Keywords:** Randomised controlled trial, Suicide prevention, Workplace, Intervention

## Abstract

**Purpose:**

In Australia and elsewhere, suicide rates among construction workers remain high. Construction workplaces are thus an important setting for targeted suicide prevention programs. This study aimed to compare suicide prevention literacy and help-seeking intentions among participants receiving face-to-face suicide prevention training, with those receiving face-to-face training augmented by a smartphone application.

**Methods:**

A two-arm randomised controlled trial of a smartphone suicide prevention intervention was conducted among construction workers in four Australian states (trial registration number: ACTRN12619000625178). All participants received face-to-face training and were randomised to the control condition (face-to-face only, *n* = 575), or *MATESmobile* condition (face-to-face + smartphone application*,* *n* = 509). Surveys administered at baseline and 3-month follow-up measured suicide prevention literacy and help-seeking intentions for personal/emotional problems and suicidal thoughts. A mixed-model repeated measures (MMRM) analysis included all 1084 randomised participants.

**Results:**

Outcomes did not differ significantly for suicide prevention literacy, nor help-seeking intentions from formal sources, informal sources outside the workplace, or no one (did not intend to seek help from anyone). However, relative to those in the control condition, those in the *MATESmobile* group showed greater increase in help-seeking intentions for emotional problems from a MATES worker/Connector (mean difference 0.54, 95% CI 0.22–0.87) and help-seeking intentions for suicidal thoughts from a workmate (mean difference 0.47, 95% CI 0.10–0.83) or MATES worker/Connector (mean difference 0.47, 95% CI 0.09–0.85).

**Conclusion:**

Results indicate that the *MATESmobile* application, together with face-to-face training, is beneficial in enhancing help-seeking intentions from MATES workers/Connectors and workmates to a greater extent than face-to-face training only. While this research provides some evidence that smartphone applications may support suicide prevention training, further research is needed.

**Supplementary Information:**

The online version contains supplementary material available at 10.1007/s00127-023-02429-9.

## Introduction

In many developed nations, construction workers have higher rates of suicide than other occupational groups [1]. This includes Australia, where male construction workers have consistently been found to die by suicide at a rate that is twice that of other employed males [2]. The suicide rate for female construction workers is also about two times the rate of female non-construction workers. While the rate among male construction workers for the period 2001–2019 was 26.9 per 100,000, the rate for female construction workers was 6.5 per 100,000 [3]. Understanding why construction workers are at high risk of suicide relative to other occupational groups has been the focus of significant research [4–7].

There are some notable contextual factors that are posited to underpin the elevated rates of suicide among construction workers. In particular, adverse psychosocial working conditions [8], including low job control and high psychological demands (the combination of which produces job strain) [9], and low social support [10], are common within the construction sector, and may increase risk of suicide. Construction work is also characterised by constant flux; workers move from job to job across different sites meaning that there are constant shifts in workplaces, colleagues, and supervisors. This transience and instability may contribute to high job insecurity [11], which is likely compounded by periods of unemployment between projects [11]. According to a meta-analysis of the effect of psychosocial job stressors on suicidality, job insecurity may increase the odds of suicide ideation by 1.91 [12], and job insecurity has been linked to an increase in death by suicide [13]. The highly male-dominated environment of construction sites, steeped in norms of traditional masculinity[14] may also contribute to increased suicide risk among construction workers given that certain masculine norms are associated with increased risk of suicidal behaviours [15, 16], and reduced help-seeking behaviours [17].

Workplaces are legislatively bound to provide safe work environments and reduce physical and psychosocial hazards. Furthermore, many workplaces have structures and resources that can support workplace suicide intervention programs (e.g. OH&S professionals, human resource personnel, union delegates). Thus, the construction workplace represents an appropriate setting for suicide prevention interventions. On this basis, Mates in Construction (MATES) was established in 2008. A charity, MATES leads an industry-based, bi-partisan, multimodal workplace-focused suicide prevention program that is delivered at construction sites and company offices.

A central component of the MATES program is a 45-min face-to-face group awareness training, General Awareness Training (GAT). For a site to be designated as ‘MATES inducted’, all workers on that worksite must participate in GAT, with an 80% training level maintained even with staff turnover. Other components of MATES build on the initial awareness raising session, and have been described elsewhere [18, 19] (also see Supplementary material).

Digital technologies are omnipresent worldwide: this is particularly characterised by almost pervasive use of smartphone technology and widespread internet connectivity. There is growing interest in the use of smartphone technology, through the use of applications (apps), to deliver or supplement interventions for different health needs [20–22]. There is some evidence of the clinical utility of smartphone applications for mental health problems; there is evidence that they are a beneficial self-management tool among those with depression [20] and may also reduce anxiety [21]. A meta-analysis of randomised controlled trials found that smartphone interventions achieved comparable impacts with face-to-face interventions in terms of mental health outcomes [22], highlighting their usefulness in assisting those with poor access to standard psychological treatment, for example among those who cannot afford treatment or those living/working remotely. The use of app-based smartphone interventions offer significant benefits by enabling participants to engage with content when and where it suits, and may facilitate engagement with groups who are otherwise difficult to reach or who are reluctant to engage with health services, including men [23]. Importantly too, smartphone interventions may be beneficial for conditions or topics that are sensitive or carry stigma, such as suicide [24]. This has been previously demonstrated in a systematic review where digital interventions, such as smartphones interventions, were associated with a reduction in suicidal ideation at follow-up [25].

Several uncontrolled evaluations have suggested that MATES face-to-face training has face validity and is an appropriate intervention for workers in the construction industry [18, 26]. There is also evidence that the MATES program is effective in reducing suicide stigma and positively shifting suicide beliefs short term[5, 7] and improving help-seeking intentions [27]. Smartphone technologies represent a potential means of complementing and reinforcing MATES core messages and enhancing suicide training outcomes. On this basis, MATES developed *MATESmobile*, an electronic platform designed to complement MATES face-to-face training (GAT), and focussing on: (1) reinforcing face-to-face training messages over time, and (2) enabling and facilitating links to mental health support where needed.

There is a need for better evidence of the effectiveness of such applications in supporting mental health. Few smartphone applications have been rigorously evaluated with comparison to a control condition, resulting in concerns around the quality of available apps [28]. Further underscoring the need to evaluate smartphone applications, it is known that problematic smartphone use may be hazardous to health [29]. The current study aimed to: (1) evaluate the implementation of *MATESmobile*; (2) assess the effectiveness of the *MATESmobile* smartphone application in complementing face-to-face training. To do this, we conducted a randomised controlled trial comparing suicide prevention literacy and help-seeking intention outcomes of participants receiving face-to-face training only, with those receiving face-to-face training and the *MATESmobile* smartphone application. It was hypothesised that relative to the face-to-face control condition, the *MATESmobile* condition would be associated with:

1. greater improvement in suicide prevention literacy,
2. increased help-seeking intentions for emotional problems,
3. increased help-seeking intentions for suicidal thoughts.

## Methods

### Trial design

This study was a two-arm randomised control trial in which participants were randomly assigned to the control or treatment condition in a 1:1 ratio. The trial was prospectively registered with the Australian and New Zealand Clinical Trial Registry (ACTRN12619000625178). Our approach was detailed in a protocol published in accordance with SPIRIT guidelines [30]. The design, conduct and reporting of this trial adhered to the Consolidated Standards of Reporting Trials (CONSORT) guidelines.

Deviations from the protocol as initially registered were principally due to the disruption caused by the COVID-19 pandemic. During fieldwork, COVID-19-related lockdown measures were implemented across Australia. In response to a slow recruitment rate, the following changes were implemented. First, the planned 6- and 12-month follow-up was dropped. Although we attempted follow-up at 6 months, low response rates meant that we were unable to use the data collected. To improve response rates, we offered incentives to participants; initially, a 1 in 5 chance to win a $50 gift-card upon survey completion, later modified to $20 voucher for all survey completions. Respondents of the baseline survey were also contacted by a research assistant to improve response rates to follow-up surveys. To understand the impact of COVID-19 on participation, we conducted a focus group with field officers. Information about help-offering behaviour information was not collected in the trial and finally, user engagement metrics were unable to be collected in the detailed form anticipated at the time of writing the protocol. Therefore, we could not match user engagement data to participants’ unique IDs and as a consequence, could not conduct a per protocol analysis. We also note that while the protocol described the collection of secondary outcomes, only primary outcomes are reported in this evaluation.

### Study setting and participants

The study was conducted at construction sites in four Australian states: New South Wales, Queensland, South Australia, and Western Australia. Participants were recruited from face-to-face training sessions at MATES sites, these were mainly sites that were managed by industry partners involved in the study. All workers attending a face-to-face training session from November 2019 to January 2020 were eligible for the study.

### Treatment

#### Control condition

All respondents in the MATES condition received face-to-face training. General Awareness Training (GAT), this 45-min face-to-face training session was provided to construction workers on-site. GAT face-to-face training presents suicide as preventable, and aims to reduce stigma and encourage help-seeking and help-offering.

#### Intervention condition (MATESmobile)

This intervention included face-to-face training as described above, plus access to the *MATESmobile* application. Once participants completed face-to-face training and were allocated to the treatment condition, they were invited to download the smartphone application. The application contained some material tailored to the respondents’ level of prior engagement with the MATES program: when an invitee first installed the app, they were presented with a brief survey that asked them to nominate whether they had completed other forms of MATES training in addition to GAT (see Supplementary material). Depending on their response, the ‘toolbox’ section of the app displayed refresher content appropriate to an individual’s level of prior engagement with the MATES program. The app contained a section titled, ‘Stories from MATES’, featuring videos focussed on recognising distress in others, connecting individuals to help, along with a lived experience video where construction workers discussed experiences of suicidal distress and return to work. The app also featured a news notification centre, a poster gallery (with downloadable content for printing), and a call button that immediately dialled the MATES support line.

Except for the toolbox content, the app content was made available to users incrementally. Videos, posters, and other materials were scheduled for release over the intervention period, and participants were contacted fortnightly (via multi-media messaging service, MMS) and advised of new content available in the app.

### Outcome measures

#### Suicide prevention literacy

Suicide prevention literacy was measured with a modified version of the face-to-face training suicide awareness questionnaire [18]. Participants were asked to indicate their agreement to four statements about suicide prevention. Their responses were ranked on a five-point Likert scale from *strongly disagree* to *strongly agree*.

The four items were combined to form a suicide prevention literacy scale. Principal components analysis (PCA) with orthogonal rotation using the Kaiser criterion identified and confirmed a single factor (eigenvalue 1.35), accounting for 57% of the variance. Cronbach’s alpha for the final scale demonstrated satisfactory internal consistency (0.74), and these items were, therefore, analysed as a single suicide prevention literacy scale.

#### Help-seeking intentions

Help-seeking intentions were measured using the General Help-seeking Questionnaire (GHSQ). GSHQ questions were modified to identify twelve different sources of help-seeking (including none, i.e., from ‘no one’), and participants were asked to rank their help-seeking intentions when experiencing either personal/emotional problems or suicidal thoughts. Intentions were ranked from *extremely unlikely* to *extremely likely* on a seven-point scale [31]*.* Average scores for emotional/mental problems and suicidal thoughts were assessed separately and classified into five categories of help-seeking sources. The MATES approach aims to encourage help-seeking from MATES workers (field officers employed by MATES to provide ongoing support to MATES sites) and Connectors (volunteer “gatekeepers” who help at-risk workers access help and support), so for this reason we distinguished between these and other sources of help. There is also some emphasis in the MATES program on help-seeking from, and help-offering to, workmates. Given this, and also given the power differential between supervisors and workers, we distinguished between work supervisors and workmates. The five categories of help-seeking were: (1) ‘formal’ sources of help (mental health professionals, doctors/GPs, and phone helplines), scores ranging from 3 to 21; (2) informal sources of help (intimate partners, relatives, friends, ministers or religious leaders and work supervisors), scores ranging from 5 to 35; (3) MATES workers/Connectors, scores ranging from 1 to 7; (4) workmates, scores ranging from 1 to 7; (5) ‘no one’ (i.e., choosing not to seek help), scores ranging from 1 to 7. The latter three categories (MATES workers/connectors, workmates, ‘no one’) were individually assessed on the basis of single items, and the classification of formal versus informal sources of help was based on the precedent of other work [32].

### Sample size

Sample size calculations were based on previous research of the primary outcomes (suicide prevention literacy) [5]. Our protocol paper specified a required sample size of 295/group (590 participants in total) [30]**.** Allowing for attrition (incomplete baseline responses and loss to follow-up), the total sample size required was estimated to be 844 participants. We had to recruit at the worksite level for this project, rather than at the individual level. As a consequence, our target sample was exceeded, with 1239 participants completing face-to-face training and being assessed for eligibility. Of the total sample, *n* = 155 were excluded, because they did not provide contact details and were unable to be randomised.

### Randomisation (allocation)

Baseline surveys were administered to respondents by MATES field officers prior to face-to-face training between November 2019 and January 2020. Baseline data collection included mobile phone numbers, and those baseline participants returning a mobile phone number were randomly allocated in blocks of 50 persons to the control or *MATESmobile* condition. A total of 1239 participants were assessed for eligibility for randomisation. Out of these, 1084 were randomised. The allocation of participants to the treatment conditions was carried out by a statistician who was external to the project, and all investigators were blinded to this process. Following randomisation, those in the *MATESmobile* condition were contacted via MMS notification and asked to download the MATES mobile application using the link provided.

### Survey

Outcomes were assessed at baseline, as well as follow-up (3 months post-intervention). At baseline, a range of sociodemographic variables were collected. These covariates included age, gender, occupation, Indigenous status, country of birth and previous MATES training. Age was classified in 10-year age groups. Occupation was coded to the two-digit level as per Australian and New Zealand Standard Classification of Occupations (ANZSCO). This was then collapsed to ANZSCO one-digit codes to provide a measure of occupational skill level ranging from 1 to 8 (highest to lowest skill). We also derived a dichotomous measure of occupational grouping from the ANZSCO 1-digit measure representing white-collar and blue-collar workers.

Baseline surveys were collected November 2019–January 2020, and follow-up data collection commenced in March 2020, and was completed in June 2020.

### Loss to follow-up

A total of 383 out of the 575 participants who were allocated to the control condition were lost to follow-up, with 192 returning follow-up surveys. Among those allocated to the *MATESmobile* condition (*n* = 509), 151 returned follow-up surveys and 358 did not. Sensitivity tests showed that baseline outcomes were not predictors of drop-out (see Table S2).

### Analytic approach

Summary measures of outcomes and sociodemographic variables were computed and compared between treatment and control groups. No formal statistical assessment of balance on baseline variables was conducted [33].

All analyses were intention-to-treat, and included all participants who were randomised, regardless of intervention received or non-participation in follow-up [30]. Mixed-model repeated measures (MMRM) analyses were used because of the ability of this approach to use all available data. This method assumes that missingness is related to observed variables in the analysis but not unobserved values, and in doing so, allows for the inclusion of participants with missing data without using potentially biased techniques, such as last observation carried forward. Degrees of freedom were estimated using the Kenward and Rogers approach [34]. The matrix estimating the variance on each occasion, and the covariance between occasions, was unstructured. The average treatment effects of *MATESmobile* were assessed by fitting an interaction between time (baseline and 3-month follow-up) and allocated intervention (control vs. *MATESmobile* intervention). Effect sizes were calculated between groups at follow-up using Cohen’s d measure of standardised mean difference.

﻿There are differing views on the appropriateness of adjusting for multiple outcomes such as with the Bonferroni correction, with some arguing for and others against their use [35]. Because the outcomes assessed in this study were specified a priori based on a program logic and included in a prospective trial registration and a published protocol, adjusting for multiple comparisons in this study is not appropriate or justified [30, 36]. However, we have conducted sensitivity analyses with Bonferroni correction at the p-level of *p* < 0.0045 (0.05 divided by 11) accepted for each set of analyses. We also carried out a further set of sensitivity analyses. Noting substantial skew in the distribution of six variables (help-seeking intentions for both suicidal thoughts and emotional problems for MATES workers/connectors, workmates and no one), we normalised residuals and estimated associations using the normalised transformations.

We assessed loss to follow-up by separately assessing the association between baseline outcomes and loss to follow-up. We also conducted a multivariate regression analysis assessing risk of loss to follow-up, including significant predictors of missing as covariates. These variables were help-seeking intentions for suicidal thoughts from formal health and MATES worker, and help-seeking intention for emotional problems from formal sources of help. Lastly, we restricted the analysis to participants who returned a follow-up questionnaire.

## Results

### Participant flow and numbers analysed

**Fig. 1:**
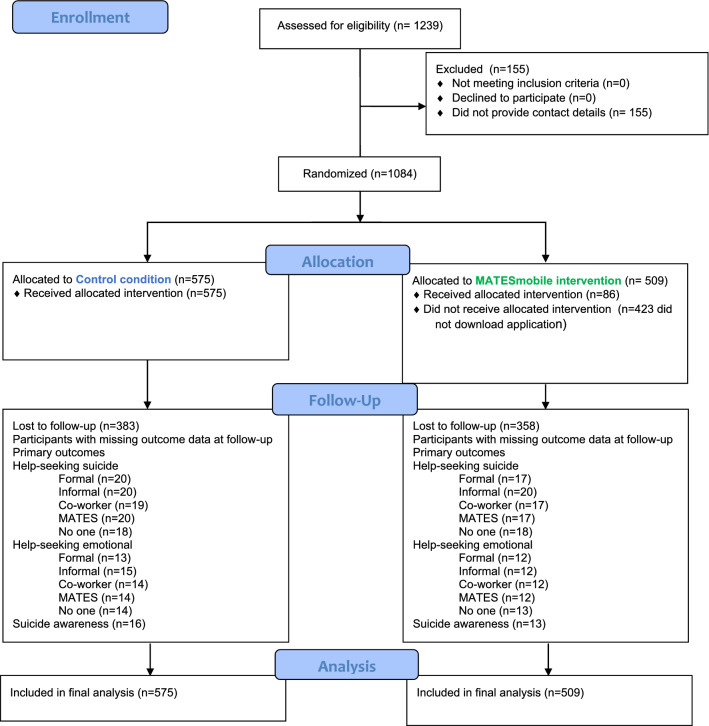
Consort flow diagram of sample

Figure 1 presents the CONSORT flow diagram of participants at each stage of the trial. All 1084 participants completing the baseline survey were randomised to either treatment arm and were included in analyses. Of the 509 participants recruited into the face-to-face + *MATESmobile* intervention, *n* = 86 downloaded the app.

### Evaluation of implementation: MATES mobile user engagement

We sought to evaluate the degree to which participants in the intervention arm participated in the intervention by recording app user engagement metrics. There were 86 active users across the period from 11 November 2019 until 31 May 2020. The number of unique users increased on the day of, and in the days after, invitations were sent. The average engagement time participants spent was 124.2 s per visit. Users spent most of their time on the toolbox section of the app, accounting for 55% of use with an average of 49.9 s. Video playing accounted for 2.7% of use, with an average of 34.8 s. The total engagement time during the first 3 months of follow-up was 7426.6 s, with an average daily use of 96.5 s.

### Evaluation of trial effectiveness

The two intervention groups were similar in terms of baseline sociodemographic characteristics, (Table 1). Most of the sample were male, born in Australia, blue collar and of low occupational skill level. The sample was young, with more than half of the respondents aged under 40 years. While most of the sample were non-Indigenous, Indigenous representation was comparable with the Australian population [37]. Just under two-thirds of respondents had not previously engaged in MATES training, with GAT being the most common type of training that respondents had previously participated in.

**Table 1:** Baseline distribution of outcomes and covariates (including missing)

	Control	MATESmobile
	(*n* = 575)	(*n* = 509)
	*n* (%)	*n* (%)
Age group (years)		
17–29	160 (27.8)	144 (28.3)
30–39	145 (25.2)	118 (23.2)
40–49	111 (19.3)	97 (19.1)
50–59	68 (11.8)	81 (15.9)
≥ 60	26 (4.5)	18 (3.5)
Missing	65 (11.3)	51 (10)
Gender		
Female	44 (7.7)	27 (5.3)
Male	488 (84.9)	452 (88.8)
Missing	43 (7.5)	30 (5.89)
Occupational skill level		
High	293 (51.0)	231 (45.4)
Low	254 (44.2)	252 (50.0)
Missing	28 (4.87)	26 (5.11)
Occupation group		
White collar	66 (11.5)	62 (12.2)
Blue collar	481 (83.7)	421 (82.7)
Missing	28 (4.87)	26 (5.11)
Indigenous status		
Indigenous Australian	19 (3.30)	14 (2.75)
Non-Indigenous Australian	490 (85.2)	453 (89.0)
Missing	66 (11.5)	42 (8.25)
Country of birth		
Australia	367 (63.8)	334 (65.6)
Other	163 (28.4)	140 (27.5)
Missing	45 (7.83)	35 (6.88)
Previous MATES training (overall)		
Yes	189 (32.9)	173 (34.0)
No	352 (61.2)	300 (58.9)
Missing	34 (5.91)	36 (7.07)
Previous GAT training		
Yes	108 (17.7)	90 (17.7)
No	434 (75.5)	383 (75.3)
Missing	33 (5.74)	36 (7.07)
Previous connector training		
Yes	51 (8.87)	51 (10.0)
No	491 (85.4)	422 (82.9)
Missing	33 (5.74)	36 (7.07)
Previous ASIST training		
Yes	14 (2.43)	11 (2.16)
No	528 (91.8)	462 (90.8)
Missing	33 (5.74)	36 (7.07)

Observed means of outcomes at baseline and follow-up are presented in Table 2 and indicate baseline comparability of groups. Table 2 also presents the within treatment arm mean differences in change between baseline and follow-up for each of the outcomes. We identified some baseline differences in mean scores of help-seeking intentions and suicide prevention literacy between the *MATESmobile* and the control group. Overall, these differences were small, but were greatest for help-seeking scores for suicidal thoughts from informal sources (23.9 vs 26.2 for the *MATESmobile* and the control groups, respectively).

**Table 2:** Observed means for outcome measures and changes from baseline to follow-up for control and *MATESmobile* treatment groups

Outcomes (score range)	Control (*n* = 575)			MATESmobile (*n* = 509)		
	Baseline	Followup	Mean difference (95% CI)	Baseline	Followup	Mean difference (95% CI)
	mean (sd)			mean (sd)		
Help-seeking intentions for suicidal thoughts^a^						
Formal help^b^ (3 to 21)	13.7 (4.48)	12.0 (4.78)	− 1.65 (− 2.62, − 0.68)	12.9 (5.03)	12.0 (5.11)	− 0.84 (− 2.05, 0.37)
Informal help^c^ (5 to 35)	26.2 (7.85)	22.8 (7.89)	− 3.45 (− 5.11, − 1.80)	23.9 (7.78)	21.7 (7.74)	− 2.22 (− 4.10, − 0.34)
MATES worker/Connector (1 to 7)	4.42 (1.75)	4.19 (1.79)	− 0.22 (− 0.59, 0.15)	4.11 (1.82)	4.44 (1.86)	0.33 (− 0.11, 0.77)
Workmate (1 to 7)	4.01 (1.74)	3.54 (1.78)	− 0.47 (− 0.84, − 0.10)	3.40 (1.74)	3.71 (1.78)	0.11 (− 0.31, 0.53)
No one (1 to 7)	2.45 (1.73)	2.36 (1.76)	− 0.09 (− 0.46, 0.27)	2.81 (1.83)	2.51 (1.64)	− 0.30 (− 0.72, 0.11)
Help-seeking intentions for personal/emotional problems^a^						
Formal help^b^ (3 to 21)	12.8 (4.37)	11.8 (4.70)	− 1.44 (− 2.37, − 0.51)	12.8 (4.37)	11.7 (4.62)	− 1.13 (− 2.18, − 0.07)
Informal help^c^ (5 to 35)	26.6 (6.91)	23.5 (7.01)	− 3.03 (− 4.50, − 1.56)	25.5 (6.14)	22.7 (6.63)	− 2.82 (− 4.33, − 1.30)
MATES worker/Connector (1 to 7)	4.53 (1.59)	4.33 (1.69)	− 0.20 (− 0.54, 0.14)	4.22 (1.62)	4.58 (1.64)	0.35 (− 0.03, 0.73)
Workmate (1 to 7)	4.10 (1.51)	4.15 (2.58)	− 0.30 (− 0.61, 0.01)	3.80 (1.52)	3.93 (1.68)	− 0.22 (− 0.60, 0.16)
No one (1 to 7)	2.58 (1.71)	2.43 (1.58)	− 0.15 (− 0.49, 0.19)	2.49 (1.59)	2.49 (1.59)	− 0.44 (− 0.83, − 0.04)
Suicide prevention literacy (4 to 20)^a^	16.8 (2.16)	17.4 (2.04)	0.61 (0.17, 1.04)	16.8 (2.02)	17.0 (2.15)	0.20 (− 0.28, 0.69)

^a^Higher scores indicate greater help-seeking intentions/awareness

^b^Formal help includes mental health professionals, doctors/GPs, and phone helplines

^c^Informal help includes intimate partners, relatives, friends, ministers or religious leaders and work supervisors

Tables 3 presents the estimated mean differences in changes in scores for outcomes from baseline to follow-up, between the two treatment groups. The difference between treatment groups in change in suicide prevention literacy was not significant. Further, there was no statistically significant difference between groups in change in scores for intentions to seek formal and informal help, nor was there a difference in change for “no one”.

**Table 3:** Estimated differences in changes from baseline to follow-up between control and *MATESmobile* treatment groups

Outcomes (score range)	Mean difference in change^a^ (95%CI) *p* value	Effect size Cohen’s *d*
Help-seeking intentions for suicidal thoughts^b^		
Formal help^c^ (3 to 21)	0.67 (− 0.28, 1.62) 0.164	0.00
Informal help^d^ (5 to 35)	0.69 (− 0.80, 2.18) 0.361	0.14
MATES worker/Connector (1 to 7)	0.47 (0.09, 0.85) 0.015	− 0.14
Workmate (1 to 7)	0.47 (0.10, 0.83) 0.012	− 0.10
No one (1 to 7)	− 0.62 (− 0.49, 0.36) 0.777	− 010
Help-seeking intentions for personal/emotional problems^b^		
Formal help ^c^ (3 to 21)	0.34 (− 0.58, 1.23) 0.480	0.03
Informal help^d^ (5 to 35)	0.18 (− 1.17, 1.54) 0.789	0.12
MATES worker/Connector (1 to 7)	0.54 (0.22, 0.87) 0.001*	− 0.15
Workmate (1 to 7)	0.21 (− 0.11, 0.53) 0.201	− 0.08
No one (1 to 7)	− 0.18 (− 0.55, 0.20) 0.355	− 0.03
Suicide prevention literacy (4 to 20)^b^	− 0.36 (− 0.81, 0.10) 0.122	0.19

^a^Difference in the average change of outcome scores from baseline to follow-up between intervention groups (control vs MATESmobile, positive scores indicating greater improvement in the MATESmobile condition)

^b^Higher scores indicate greater help-seeking intentions/awareness

^c^Formal help includes mental health professionals, doctors/GPs, and phone helplines

^d^Informal help includes intimate partners, relatives, friends, ministers or religious leaders and work supervisors

*Remains significant after Bonferroni correction for multiple testing (accepted *p* value after Bonferroni correction is *p* < 0.0045)

The change from baseline to follow-up for intentions to seek help from a MATES worker/Connector for suicidal thoughts was a mean difference of 0.47 higher for the *MATESmobile* group than for the control group (95%CI 0.09, 0.85). This corresponded to an effect size of − 0.14. Similarly, there was a greater positive change in intentions to seek help for suicidal thoughts from a workmate in the *MATESmobile* group (0.47, 95% CI 0.10–0.83), an effect size of − 0.10.

When considering help-seeking intentions for emotional problems, those in the *MATESmobile* group displayed a greater increase in help-seeking intentions from a MATES worker/Connector (0.54, 95% CI 0.22–0.87), corresponding to an effect size of − 0.15 (95%CI − 0.37, 0.08). This association remained after testing for multiple comparisons with the Bonferroni correction, however, the associations for help-seeking intentions for suicidal thoughts were not significant after testing for multiple comparisons.

Sensitivity analyses carried out on the six skewed outcomes were consistent with the main analyses in terms of direction, but produced smaller *p* values (see Supplementary Table S1). Our findings did not substantially change across analyses restricted to participants who participated at follow-up (see Table S3).

## Discussion

Despite low uptake of the *MATESmobile* intervention, our second hypothesis was partially supported with evidence that the *MATESmobile* condition (*MATESmobile* application + face-to-face training) was more effective than face-to-face training alone in increasing respondents’ intentions to seek help from MATES workers/Connectors for emotional problems. There was also evidence that the *MATESmobile* condition was more effective than face-to-face training alone in increasing respondents’ intentions to seek help for suicidal thoughts from MATES workers/Connectors and workmates, however, we note that these associations were not significant when the Bonferroni correction was imposed. We also note that the effect sizes indicate that the differences between the two groups were small for each of the significant results.

There was no evidence that the *MATESmobile* treatment condition changed intentions to seek other sources of help, either informal or formal, nor was there support for the first hypothesis, with no evidence that *MATESmobile* improved suicide prevention literacy more than MATES face-to-face training alone.

While effect sizes were small, overall, these results are encouraging, with some evidence that face-to-face training in conjunction with the *MATESmobile* application reinforces MATES messages and improves intentions to seek help. The positive effects are consistent with the main message of MATES training for workers, which emphasises the role of Connectors as conduits of help. Another main message of MATES is in relation to the role of workmates as a source of help, and the result for intentions to seek help from a workmate for suicidal thoughts (although not significant after testing for multiple comparisons) aligns with this messaging. The centrality of workmates and Connectors to the MATES program is articulated in the program logic, which gives further confidence in the meaningfulness of these findings [36]. It is not clear whether the lack of significant differences in help-seeking intentions for formal and informal sources of help was due to a lack of power or program ineffectiveness on these domains. Nonetheless, finding a detectable effect in intention-to-treat analysis suggests that either a much larger effect may have occurred among those who engaged with the intervention, or that there was transmission of messages within the intervention group that was unrelated to the application. The latter is highly unlikely, and the results suggest that the application may be highly effective at reinforcing messages among users who engage at least minimally with the app.

There is growing evidence of the utility of digital technologies including smartphone applications and web-based interventions in supporting the treatment and management of depressive symptoms [38, 39]. Meta-analyses and systematic reviews have also reported promising evidence of the effectiveness of smartphone applications for symptoms of depression and anxiety [20–22]. The tentative evidence that the *MATESmobile* smartphone application improved intentions to seek help from MATES workers/Connectors comports with these studies.

We also note that help-seeking intentions from formal and informal sources of help for both suicidal thoughts and emotional problems decreased at follow-up for the intervention and control groups. This may be due to some broader societal or construction related factors that impacted on help-seeking intentions for both groups. As noted, follow-up for both treatment groups occurred as the country was experiencing the first wave of the COVID-19 pandemic. It is possible that this impacted on help-seeking intentions for both groups.

There are several key limitations of this analysis. First, the pandemic represented a major disruption to our research program. The impact of COVID-19 on the operation of construction sites varied across state jurisdictions, with some sites closed for periods of time, some operating with reduced numbers of workers and others having restrictions imposed in terms of access, interaction and engagement. While the effects of the pandemic impacted many elements of the research program including implementation of the intervention, the disruption was most starkly revealed by the attrition of participants—this was substantial, despite our success in recruiting more participants than defined in our protocol. The sample drop-out reduced power to detect an intervention effect and makes it difficult to ascertain the extent to which the intervention was impacted by implementation failure related to COVID-19, program ineffectiveness, or the program not sufficiently meeting the needs of participants. Second, uptake of the app was limited, with only 17% of those allocated to the *MATESmobile* intervention group downloading the application. Application user analytic data were also limited, so we were unable to assess app usage and engagement according to treatment allocation. It may be the case that the app is primarily beneficial to the subgroup of the population most vulnerable to suicidal distress, in which case delivery as an indicated prevention intervention may be more appropriate than use of the app as a universal prevention intervention. Further research is needed to examine the utility of the application before it is universally distributed. Given the low response rate at follow-up, the low uptake of the app, and limited analytic data, the generalisability of these results is unclear. This said, a strength of our approach is that the app usage in this study likely matched real world patterns [40].

While these results are promising, there is a need for further research to assess the extent to which the *MATESmobile* application may improve help-seeking from other sources to a greater extent than face-to-face training alone. We also note that help-seeking intentions do not necessarily correspond with reduced suicide risk. Help-seeking intentions are one relatively distal link in the chain of steps between the point of crisis and suicide. Following a crisis event, help-seeking intentions precede help-seeking behaviours, and these in turn precede health service access and delivery. The extent to which services meet individual needs is an additional unknown. Research is needed to understand associations between these constructs, and their ultimate efficacy in preventing suicide.

## Conclusions

Overall, this randomised controlled trial suggests that the *MATESmobile* application improves help-seeking intentions for emotional problems from MATES workers/Connectors to a greater extent than face-to-face training only. While there was also some weak evidence that the application improved help-seeking for suicidal thoughts from workmates and MATES workers/Connectors, there was no evidence that it improved help-seeking from formal or informal sources. Overall, these results suggest that the *MATESmobile* application may be of modest benefit in supporting MATES face-to-face general awareness training, however, further evidence is needed.

## Supplementary Information

Below is the link to the electronic supplementary material.

Supplementary file1 (DOCX 26 KB)

## Data Availability

The participants of this study did not give written consent for their data to be shared publicly, so due to the sensitive nature of the research supporting data is not available.
