# Supplementary material for: A blended face-to-face and smartphone intervention to improve suicide prevention literacy and help-seeking intentions among construction workers: a randomised controlled trial
Source: Soc Psychiatry Psychiatr Epidemiol. 2023 Feb 9;58(6):871–81. doi: 10.1007/s00127-023-02429-9 (PMC9909155; doi:10.1007/s00127-023-02429-9)
Supplement: Supplementary file 1 — Supplementary file1 (DOCX 26 KB) [file 127_2023_2429_MOESM1_ESM.docx]

**Supplementary Material: Effectiveness of a blended face-to-face and smartphone intervention for suicide prevention in the construction industry: a randomised controlled trial**

**Material Sa: Description of the MATES program**

[Mates in Construction (MATES)](https://mates.org.au/about-us) was established in Queensland (Australia) in 2008 by the Building Employees Redundancy Trust with the express aim to prevent suicide in the construction industry. A charity, MATES leads an industry-based, multimodal workplace-focused suicide prevention program that is delivered at construction sites and company offices.

A central component of the MATES program is 45-minute face-to-face group awareness raising training, referred to as General Awareness Training (GAT), which is provided to all construction workers on site. GAT face-to-face training presents suicide as preventable, and aims to reduce stigma and encourage help-seeking and help-offering behaviour. For a site to be designated as ‘MATES inducted’, all workers on that worksite must participate in GAT, with an 80% training level maintained even with staff turnover. Face-to-face training is also a component of a training program ﻿specifically for apprentices. There are other components of MATES that build on the initial awareness raising session, and have been described elsewhere (Gullestrup, Lequertier & Martin 2011; Martin et al. 2016).

These include: *“Connector training”* for volunteer “gatekeepers” (Connectors help at-risk workers access help via a trained worker, MATES Field Officer, Case Manager or other local community supports); and Applied Suicide Intervention Skills Training (ASIST), a 16-hour Livingworks suicide intervention training program available to interested volunteers. The MATES program also includes *Field Officers*, who are employed directly by MATES to provide ongoing support to MATES sites through regular site visits, establishing and maintaining relationships with workers on-site, and debriefing Connectors; and *Case managers*, who are also MATES employed and qualified to assist troubled workers with a plan to effectively address their problems and concerns. This could include connecting workers with such services as their EAP, financial counselling, mental health services, drug and alcohol services, grief counselling, or family and relationship counselling.

Gullestrup, J, Lequertier, B & Martin, G 2011, ‘MATES in construction: Impact of a multimodal, community-based program for suicide prevention in the construction industry’, *International Journal of Environmental Research and Public Health*, vol. 8, no. 11, pp. 4180–4196.

Martin, G, Swannell, S, Milner, A & Gullestrup, J 2016, ‘Mates in Construction Suicide Prevention Program: A Five Year Review’, *Journal of Community Medicine & Health Education*, vol. 6, no. 4, pp. 1–8.

**Table S1. Estimated difference in mean change for skewed outcomes after normalising transformation.**

| **OUTCOMES** | **Mean difference in change (95%CI) p-value** |
| --- | --- |
| ***Help-seeking intentions (suicidal thoughts)*** |  |
| *MATES worker/Connector (1 to 7)* | 0.227 (0.056, 0.398) 0.009 |
| *Workmate (1 to 7)* | 0.290 (0.067, 0.513) 0.011 |
| *No one (1 to 7)* | 0.001 (-0.020, 0.017) 0.908 |
| ***Help-seeking intentions (emotional problems)*** |  |
| *MATES worker/Connector (1 to 7)* | 0.235 (0.098, 0.372) 0.001 |
| *Workmate (1 to 7)* | 0.119 (-0.046, 0.282) 0.156 |
| *No one (1 to 7)* | 0.006 (-0.003, 0.016) 0.195 |

**Table S2. Participants’ risk of dropping out from the study according to baseline outcomes.**

| **Baseline outcome** | **Univariate analysis** | **Multivariate analysis** |
| --- | --- | --- |
| ***Help-seeking intentions (suicidal thoughts)*** | **RR (95%CI) p-value** | **RR (95%CI) p-value** |
| *Formal help* | 0.98 (0.98, 1.00) 0.004 | 0.99 (0.97, 1.00) 0.096 |
| *Informal help* | 1.00 (0.99, 1.01) 0.725 |  |
| *MATES worker/Connector* | 0.97 (0.95, 1.00) 0.025 | 0.99 (0.95, 1.02) 0.420 |
| *Workmate* | 1.00 (0.97, 1.02) 0.841 |  |
| *No one* | 1.02 (0.99, 1.04) 0.140 |  |
| ***Help-seeking intentions (emotional)*** |  |  |
| *Formal help* | 0.99 (0.98, 1.00) 0.013 | 1.00 (0.99, 1.02) 0.752 |
| *Informal help* | 1.00 (1.00, 1.01) 0.970 |  |
| *MATES worker/Connector* | 0.98 (0.96, 1.01) 0.150 |  |
| *Workmate* | 1.01 (0.98, 1.04) 0.444 |  |
| *No one* | 1.02 (1.00, 1.04) 0.072 |  |
| ***Suicide prevention literacy (4 to 20)*** ^‡^ | 0.99 (0.97, 1.01) 0.239 |  |

**Table S3.** **Estimated differences in changes from baseline to follow-up between control and *MATESmobile* treatment groups for participants who were not lost to follow-up (n=192 for control, n=151 for MATESmobile)**

| **OUTCOMES** | **Mean difference in change**^¶^  **(95%CI) p-value** | **Effect size Cohen’s d** |
| --- | --- | --- |
| ***Help-seeking intentions for suicidal thoughts***^‡^ ***(score range)*** |  |  |
| *Formal help* ^∏^  *(3 to 21)* | 0.79 (-0.23, 1.80) 0.129 | 0.00 |
| *Informal help* ^§^ *(5 to 35)* | 1.03 (-0.58, 2.63) 0.209 | 0.14 |
| *MATES worker/Connector (1 to 7)* | 0.55 (0.14, 0.97) 0.009 | -0.13 |
| *Workmate (1 to 7)* | 0.56 (0.16, 0.96) 0.006 | -0.11 |
| *No one (1 to 7)* | -0.19 (-0.70, 0.31) 0.456 | 0.15 |
| ***Help-seeking intentions (emotional)*** ^‡^ |  |  |
| *Formal help* ^∏^  *(3 to 21)* | 0.33 (-0.63, 1.29) 0.502 | 0.03 |
| *Informal help* ^§^ *(5 to 35)* | 0.25 (-1.21, 1.72) 0.735 | 0.12 |
| *MATES worker/Connector (1 to 7)* | 0.56 (0.21, 0.90) 0.002 | **-**0.15 |
| *Workmate (1 to 7)* | 0.10 (-0.25, 0.44) 0.583 | -0.08 |
| *No one (1 to 7)* | -0.29 (-0.72, 0.15) 0.197 | -0.03 |
| ***Suicide prevention literacy (4 to 20)*** ^‡^ | -0.43 (-0.93, 0.08) 0.096 | 0.20 |

^¶^ Difference in the average change of outcome scores from baseline to follow-up between intervention groups (control vs *MATESmobile, positive scores indicating greater improvement in the MATESmobile condition*).

^‡^ Higher scores indicate greater help-seeking intentions/awareness.

^∏^ Formal help includes mental health professionals, doctors/GPs, and phone helplines.

^§^ Informal help includes intimate partners, relatives, friends, ministers or religious leaders and work supervisors
